# Rapid Non-Destructive Quantification of Eugenol in Curdlan Biofilms by Electronic Nose Combined with Gas Chromatography-Mass Spectrometry

**DOI:** 10.3390/s20164441

**Published:** 2020-08-09

**Authors:** Lu Han, Jingyi Zhu, Xia Fan, Chong Zhang, Kang Tu, Jing Peng, Jiahong Wang, Leiqing Pan

**Affiliations:** 1 College of Food Science and Technology, Nanjing Agricultural University, Nanjing 210095, China; hanlu@njau.edu.cn (L.H.); 2018108045@njau.edu.cn (J.Z.); fanxia@njau.edu.cn (X.F.); zhangchong@njau.edu.cn (C.Z.); jpeng@njau.edu.cn (J.P.); 2 College of Light Industry and Food Engineering, Nanjing Forestry University, Nanjing 210037, China; njfuwjh@njfu.edu.cn

**Keywords:** curdlan, biofilm, eugenol, electronic nose, GC-MS, prediction model

## Abstract

Eugenol is hepatotoxic and potentially hazardous to human health. This paper reports on a rapid non-destructive quantitative method for the determination of eugenol concentration in curdlan (CD) biofilms by electronic nose (E-nose) combined with gas chromatography-mass spectrometry (GC-MS). Different concentrations of eugenol were added to the film-forming solution to form a series of biofilms by casting method, and the actual eugenol concentration in the biofilm was determined. Analysis of the odor collected on the biofilms was carried out by GC-MS and an E-nose. The E-nose data was subjected to principal component analysis (PCA) and linear discriminant analysis (LDA) in order to establish a discriminant model for determining eugenol concentrations in the biofilms. Further analyses involving the application of all sensors and featured sensors, the prediction model-based partial least squares (PLS) and support vector machines (SVM) were carried out to determine eugenol concentration in the CD biofilms. The results showed that the optimal prediction model for eugenol concentration was obtained by PLS at R^2^_p_ of 0.952 using 10 sensors. The study described a rapid, non-destructive detection and quantitative method for determining eugenol concentration in bio-based packaging materials.

## 1. Introduction

Curdlan (CD) is a water-insoluble extracellular polysaccharide with the formula (C_6_H_10_O_5_)_n_ produced by bacteria, such as Rhizobiaceae *(Alcaligenes faecalis*) [1]. It is characterized by thermal gelation and non-toxic properties and is widely used in the food industry [2]. In addition, the excellent film-forming and biological characteristics of CD have gradually attracted attention. Currently, CD is being used as raw material for the production of bio-based films [3,4,5,6,7]. In view of safety, additives in bio-based food packaging tend to be natural compounds instead of synthetic reagents in recent years.

Eugenol (4-allyl-2-methoxyphenol) is a natural oily liquid that has been widely used in pharmaceuticals, cosmetics and food due to its widespread abundance and low cost [8,9,10]. Eugenol has broad-spectrum antibacterial properties and high antioxidant activities. Incorporating this natural compound into biofilms helps protect against certain deteriorating reactions. It has for this purpose been used considerably in active packaging for food preservation. At present, the available papers mainly report the formulation, performance and release rules of biofilms with eugenol [11,12,13], but there are few studies carried out to determine their safety status or toxicity when used in or incorporated into bio-based membranes. Although eugenol is a generally recognized as safe (GRAS) substance by the US Food and Drug Administration (FDA), it can cause increased generation of tissue damaging free radicals when used at high concentrations [14]. For example, Material Safety Data Sheet (MSDS) shows that eugenol has carcinogenic and mutagenic effects in mice. Evidence from the literature shows that eugenol is hepatotoxic and can be hazardous to human health. Also, reports from case studies indicate that eugenol oil can cause aspiration pneumonia and coma, renal failure, and disseminated intravascular coagulation [15]. According to the Food and Agriculture Organization (FAO), the acceptable daily intake of eugenol is 2.5 mg kg^−1^. It is stated in the GB9685-2016 that eugenol, as an additive of packaging materials, cannot be easily detected because of its specific migration limit (SML) requirements. However, there are no generally acceptable standard procedures to specify its addition limit and method of detection.

A variety of qualitative and quantitative methods have been reported for the detection of eugenol in different matrices, including gas chromatography-mass spectrometry (GC-MS) in fish [16,17], high performance liquid chromatography (HPLC) in smoked food samples [18] and electrochemical method in different real samples [19,20]. Although these chromatographic methods have been widely used for the analyses of complex and multicomponent samples, the cost implications of instrument operation and maintenance is high. Furthermore, analyses of samples usually require complex pretreatments and long running times, making these techniques unsuitable for rapid and cost-effective detection and quantitation of samples. Alternatively, the electrochemical method has advantages of simple operation, fast response speed and high sensitivity, and the limit of detection can be as low as 0.1 μM (S/N = 3) [14]. It has been used to determine eugenol concentration in curry powder, perfume and capsule samples, etc. However, the manufacturing process of the electrochemical sensor is complicated, selective and it has a short service life, especially samples which generally need to be prepared in liquid form for testing. All the above analytical methods can cause damage to samples, so they are not suitable for quality testing of a large number of samples. Therefore, the development of a non-destructive analytical method for the determination of eugenol concentration will be more suitable for practical applications.

In recent years, non-destructive testing technology has been receiving more attention as an emerging technology. The E-nose technology is designed to simulate the olfactory system of mammals by detecting the odor status of a specific location in real time through gas sensing array and response pattern [21]. It has attracted attention due to its advantages of simple sample processing, short response time, good recognition effect and inherent non-damage analysis [22]. E-nose technology has been widely applied in many fields such as environmental monitoring [23,24], food industries [25,26] and medical areas [27]. It has also been used to establish a classification and quantitative model of determining formaldehyde concentration in squids. The accuracy of the model is verified with R^2^ of 0.9266 in a cross-validation process [28]. Eugenol has strong clove aroma and a slightly spicy aroma. The concentration of its volatile aroma has been reported to correlate positively with its concentration. Therefore, the E-nose technology can be used to detect and provide quantitative information about the odor properties of eugenol according to its volatility without any pretreatments. The method can be used to establish a “fingerprint” pattern of eugenol concentration rapidly in a non-destructive way.

In view of the foregoing, the aim of this study was to develop a rapid non-destructive and quantitative method for detecting eugenol concentration in CD biofilms based on E-nose technology. This method will hopefully be useful for screening a large number of samples and serve as reference for guaranteeing food safety during product packaging and storage. The methodical approach entailed the preparation of a series of CD active biofilms with different eugenol concentrations by casting method. In order to verify the packaging efficiency of eugenol, the actual eugenol concentration in the biofilms was measured. Secondly, the concentration of eugenol in the different, volatile components of the biofilm samples was analyzed by gas chromatography-mass spectrometry (GC-MS) and the electronic nose (E-nose). Thereafter, qualitative differentiation models of eugenol concentration in the biofilms were established based on principal component analysis (PCA) and linear discriminant analysis (LDA). In addition, loading analysis (LA) and successive projection algorithm (SPA) were carried out to screen the featured sensors. Finally, partial least squares (PLS) and support vector machines (SVM) were applied to find an appropriate prediction model for eugenol concentration in CD biofilms.

## 2. Materials and Methods

### 2.1. Materials

CD (70–80 kDa) was purchased from Kirin Holdings Company (Tokyo, Japan). Chemicals such as eugenol, glycerol, Tween 80, lactic acid and methanol at analytical grade purity were purchased from Yuanye Biological Co., Ltd. (Nanjing, China).

### 2.2. Biofilms Preparation

Biofilms were prepared using a casting technique. To obtain a polymer concentration of 4% (w/v), CD powder and distilled water were mixed at room temperature. Glycerol (0.3 g/g CD), Tween 80 (0.2 g/g CD), and eugenol were then added to the film-forming solution (FFS). FFS containing different amounts of eugenol were prepared after the addition of (5 mg/g CD (E-5), 10 mg/g CD (E-10), 20 mg/g CD (E-20), 30 mg/g CD (E-30), 40 mg/g CD (E-40), 50 mg/g CD (E-50) and 60 mg/g CD (E-60) eugenol). The FFS devoid of eugenol was used as blank film (E-0). The FFSs were mixed using a magnetic stirrer for 30 min after which the pH of each solution was adjusted to 4 with lactic acid. The FFSs were all homogenized for 2 min, then poured into a polytetrafluoroethylene plate (100 °C) and dried in a tank for 24 h at 25 °C and 50% relative humidity (RH) to produce biofilms containing different amounts of eugenol.

### 2.3. Quantitative Determination of Eugenol in the Biofilms

The solubility of eugenol in water is 2.46 g/L (slightly soluble). It is volatile at high temperatures. In order to account for any possible loss of eugenol during the process of film formation, it was necessary to determine the concentration of eugenol in the prepared biofilms.

The eugenol concentration in each biofilm was determined according to the previous literature with some modifications [29,30]. The film sample (0.5 g) was immersed in 25 mL of methanol and then subjected to ultrasound-assisted extraction at 40 °C for 4 hours for complete extraction of eugenol. The same operation was performed for the extraction of eugenol from the blank film (E-0). The absorbance of the sample was measured at 282 nm in a UV–Vis spectrophotometer (UV-1800, Shimadzu, Japan) [13]. The absorbance difference between the sample and control measurements for all experiments was correlated with the corresponding standard calibration curves in order to determine the concentration of eugenol in the biofilms.

### 2.4. E-Nose Analysis

A PEN3 E-nose was used to analyze the odor of biofilm samples. The system is a product of Win Muster Air-sense (WMA) Analytics Inc. (Schwerin, Germany), and it consists of a sampling apparatus, a detector unit with an array of 10 different metal oxide sensors (W1C, W5S, W3C, W6S, W5C, W1S, W1W, W2S, W2W and W3S) and pattern recognition software for data recording and analysis [31,32]. The biofilm samples were placed in an experimental beaker and sealed with tinfoil. The beaker was pre-heated in the oven for 10 min at 40 °C before the start of the experiment in order to equilibrate the air in the bottle. Clean, dry air was used as the carrier gas at a flow rate of 400 mL/min in the E-nose detection, and the odor data were collected for 60 s. The odor data of 10 samples prepared from each biofilm with known eugenol concentration were collected for E-nose analysis.

### 2.5. GC-MS Analysis

Each biofilm sample (0.5 g) in a glass vial was heated at 90 °C for 10 min. The volatile compounds were adsorbed on the solid phase micro extraction (SPME) column by headspace (HS) at 45 °C for 30 min and then desorbed in the injection phase of the 7890A-5975C GC-MS (Agilent Technologies, Palo Alto, USA), which was run for 5 min at 250 °C. The gas chromatographic separation was performed on an HP-5M capillary column (30 m × 0.25 mm × 0.25 µm) coupled with a mass spectrometer in the electron ionization (EI) mode (70 eV). Helium (of 99.999% purity) was used as the carrier gas at a constant flow rate of 1.0 mL/min. The GC was run under isothermal and programmed temperatures as described subsequently. At the beginning, the column was held at 40 °C for 1 min and then raised from 40 °C to 150 °C at 6 °C/min. Next, the temperature was raised from 150 °C to 240 °C at the rate of 7 °C/min and then maintained at 240 °C for 3 min.

### 2.6. Statistical Analysis and Modeling

Ten samples of each concentration of biofilm were collected for E-nose measurement while all other experiments were carried out in triplicates. Data were analyzed by the least significant difference at *p* < 0.05 with SAS software (Version 9.2; SAS Institute; Cary, NC; 2006; USA). GC-MS detected volatile compounds were identified by a National Institute of Standards and Technology (NIST) search (NIST 2008).

For the statistical analysis of E-nose, the data at 60 s was extracted as the eigenvalue obtained from 10 sensors responses during the detection and the radar graph was used to monitor changes in sensor response values to biofilms containing different eugenol concentrations. PCA and LDA were carried out for the qualitative and semi-quantitative analyses of E-nose data using MATLAB (R2010b) and Win Muster v.1.6.2 pattern software, respectively. Furthermore, LA and SPA were carried out to screen the featured sensors. The PLS and SVM algorithms were run in MATLAB with PLS-Toolbox 5.0 (Eigenvector Research, Inc., Wenatchee, WA, USA), and applied to appropriate models based on all and feature sensors. The models obtained from the application of different modeling methods and sensors were compared to select the optimal prediction model at each eugenol concentration. Correlation coefficient of prediction (R^2^_p_), root mean square error of prediction (RMSEP) and residual predictive deviation (RPD) were used as evaluation indices of modeling effect. Generally, a good model should have high values of R^2^_p_ (close to 1) and RPD (> 3), low values of RMSEP (close to 0), indicating that the model has good stability and strong predictive ability [33,34,35,36].

## 3. Results and Discussion

### 3.1. Quantitative Determination of Eugenol in the Biofilms

**Figure 1:**
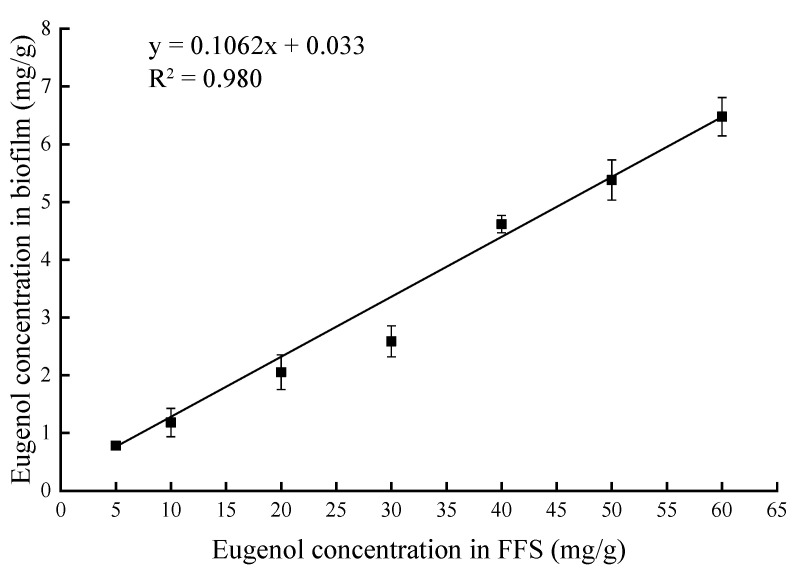
Linear concentration relationship between eugenol concentration in film-forming solution (FFS) and eugenol concentration in biofilm.

The weight of a biofilm formed was about 6.3% of the FFS. The weight loss was apparently due to the loss of water and the volatilization of active compounds. The concentration of eugenol in each biofilm sample was calculated from the standard calibration curve and shown in Table 1. It was observed that the concentration of eugenol in the biofilm increased linearly with increasing amounts of eugenol added to the FFS. The linear relationship was equivalent to R^2^ of 0.980 as shown in Figure 1. For ease of description, the eugenol concentration in FFS will be used to describe the corresponding biofilm in subsequent sections of this article. 

### 3.2. GC-MS Analysis

**Figure 2:**
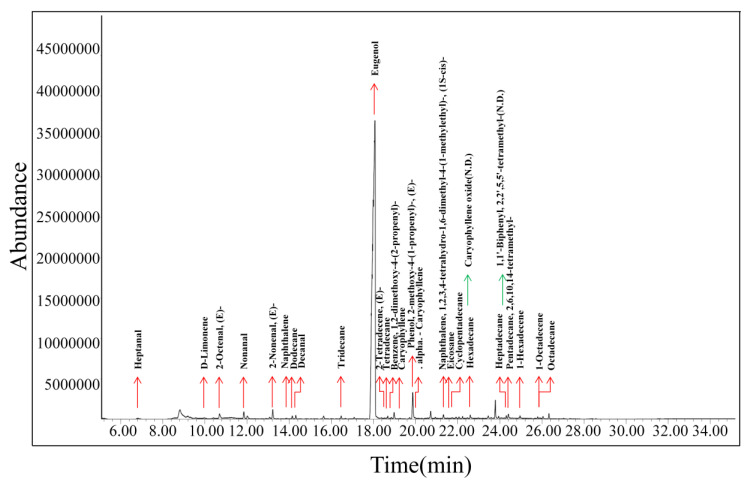
The gas chromatography-mass spectrometry (GC-MS) spectrum of volatile compounds (VOC) released by E-5 biofilm.

**Figure 3:**
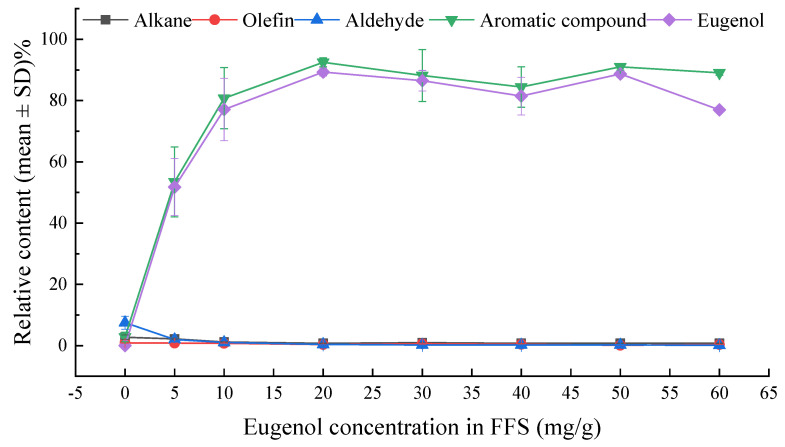
The relative concentration of alkanes, olefins, aldehydes, aromatic compounds and eugenol in different eugenol- curdlan (CD) biofilms.

There are few reports in the literature on the volatile compounds in biofilms. HS-SPME-GC-MS was applied to determine the diversities of volatile constituents in the biofilm samples with different eugenol concentrations and to validate the results of E-nose experiments. The GC-MS spectrum of volatile compounds released by the E-5 biofilm is shown in Figure 2. Twenty-seven compounds were identified, comprising nine (9) alkanes, eight (8) olefins, five (5) aldehydes and five (5) aromatic compounds (Table 2). With the increasing eugenol concentration in the biofilms, the species of volatile components did not vary significantly. The aromatic compounds with eugenol as the main component had an obvious growth trend in low concentration films. However, the relative concentration of eugenol reached a higher level from E-20, and it tends to be stable in the biofilms with higher eugenol concentrations due to saturation effect. As a result, the relative concentrations of alkanes, alkenes and aldehydes were reduced. The change trend of alkanes, alkenes, aldehydes, aromatic compounds and eugenol in different eugenol biofilms are shown in Figure 3. Among them, dextroterpene diene, 2-tetradecene, 1-hexadecene, heptanal and decanal gradually decreased or even disappeared as the concentration of eugenol in the biofilm increased.

### 3.3. E-nose Analysis

#### 3.3.1. Radar Graph

**Figure 4:**
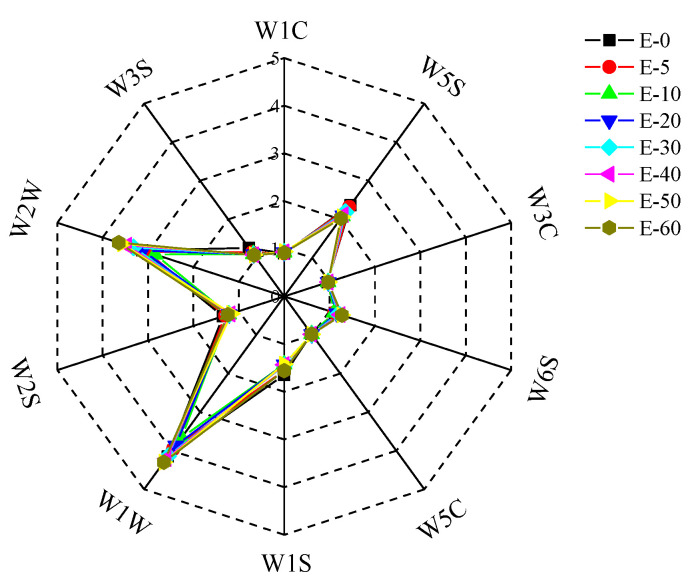
Radar graph of eight biofilms with different eugenol concentrations.

Different sensors had different responses to volatile substances in biofilms with different eugenol concentrations. The radar graph in Figure 4 shows the difference in E-nose response values of biofilm samples with different eugenol concentrations. The dots with different colors in the figure represent biofilms with different eugenol concentrations, the solid line represents 10 sensors and the dotted line represents the response value of the sensor. With increased eugenol concentration, the differentiation degree of biofilms increased. The changes in W5S, W1W and W2W were noticeable while W6S, W1S, W2S and W3S showed no significant changes. In contrast, the W1C, W3C and W5C showed no responses. The W2W sensor was sensitive to aromatic hydrocarbons in an increasing manner while W1S and W3S showed sensitivity to alkanes in a decreasing manner. These results were consistent with the results of the GC-MS analysis.

#### 3.3.2. PCA and LDA Analysis

**Figure 5:**
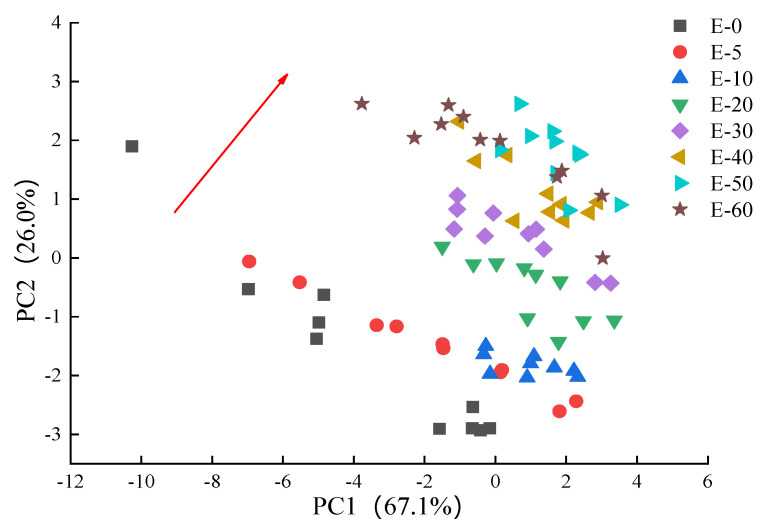
Principal component analysis (PCA) results of biofilms with different eugenol concentrations.

Pattern recognition can provide direct and easily understood qualitative and semi-quantitative data. PCA is a projection approach used for reducing the dimensionality of data, calculating a number of variables that best describe the differences between the samples and are arranged according to the contribution rates (called principal components (PCs)) [32,37]. In order to determine the effectiveness of the E-nose technique to detect and differentiate biofilms of different eugenol concentrations, PCA was used to process the data of 10 different sensors in order to reduce the complexity of the data. As shown in Figure 5, the contribution rate of the first principal component (PC1) was 67.1% and that of the second principal component (PC2) was 26%. The result showed that biofilms with large differences in eugenol concentrations could be effectively discriminated, but there were overlaps between adjacent samples, especially the E-60 biofilm that could not be separated from other biofilms. Because PCA only fits a handful of samples at a time, when the number of biofilm samples to be measured increases, the biofilm samples with different eugenol concentrations will become crowded and not easily distinguished especially in adjacent samples in the PCA chart [38]. However, in general, the distribution trend of the sample membranes with different concentrations was shown with the red arrow.

**Figure 6:**
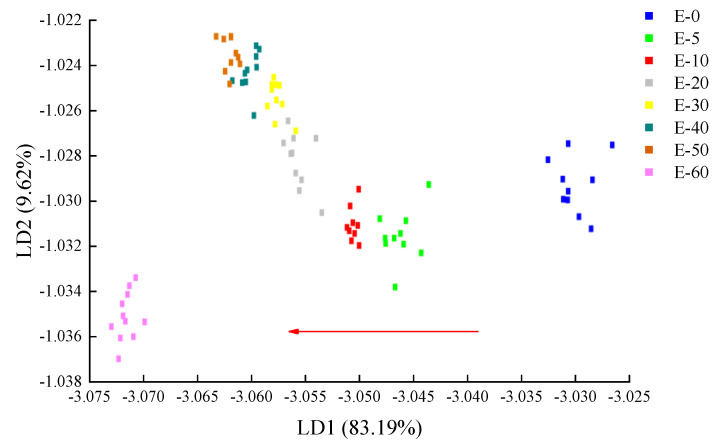
Linear discriminant analysis (LDA) results of biofilms with different eugenol concentrations.

To further investigate E-nose data, a qualitative discriminant model was established using LDA to quickly discriminate biofilms with different eugenol concentrations. LDA is a statistical method that can determine which group the sample belongs to by maximizing the variance between categories and minimizing the variance within categories [38]. As shown in Figure 6, the total LDA contribution of eugenol biofilm was 92.81%, which was slightly lower than the PCA analysis (93.1%). However, in the LDA analysis, the degree of dispersion between the active biofilm samples was greater than that of the PCA analysis, which was effectively used to differentiate biofilms of different eugenol concentrations. Based on eugenol concentration in the biofilms, the samples were distributed from large to small along the LD1 side as indicated with the red arrow. The above results provide a reference for further differentiation.

#### 3.3.3. Featured Sensor Screening

The radar graph shows that some sensors have little contributions to response discrimination. This implied that optimization of the sensor array did not only effectively eliminate useless and abnormal sensors, it also reduced the amount of data, improved the accuracy and speed of operation and reduced the production cost of the system. This study adopted the LA and SPA to optimize the sensor array in order to obtain the feature sensors for the determination of eugenol concentration in the biofilms.

**Figure 7:**
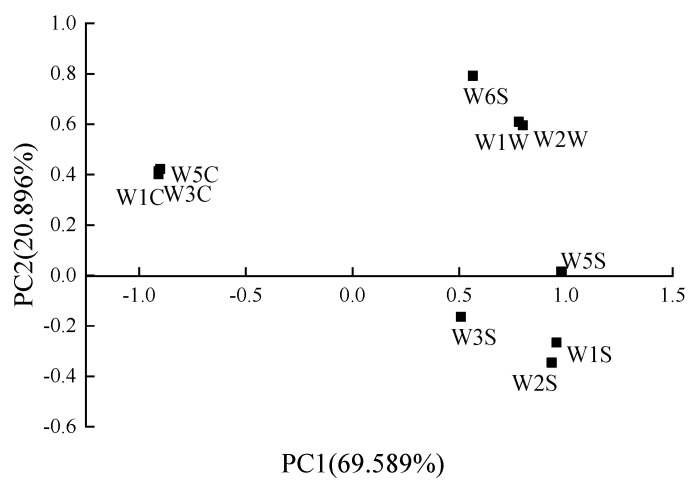
Loading analysis (LA) of sensor response values of biofilms with different eugenol concentrations.

Figure 7 shows the load analysis diagram of the sensor response values of biofilm samples of different eugenol concentrations. The diagram was used to evaluate the contribution value of the arrays of sensors used to differentiate the odor changes of the biofilms containing different eugenol concentrations. According to the figure, except for sensors of W1C, W3C and W5C, the scores of other sensors on PC1 were more than 0.5. The contribution of sensors of W5S, W1S and W2S on PC1 were greater than that of PC2 (with values greater than 0.9), while the contribution of sensors of W6S, W1W and W2W on PC2 were greater. Although the contribution rate of PC1 was higher than that of PC2, the contribution rate of PC2 was not lower, so the contribution of PC1 and PC2 should be considered comprehensively. According to the load diagram of the sensor response values of biofilms with different eugenol concentrations, the contributions of W1C, W3C and W5C sensors on PC1 and PC2 was little, implying their ineffectiveness in identifying the odor changes of biofilms with different eugenol concentrations. For the remaining sensors, the load factor scores of W6S, W1W, W2W and W5S, W1S, W2S were comparable, indicating a strong correlation between the sensors and the similarity of their recognition effects. There might be data overlap and further optimization and screening may be required.

**Figure 8:**
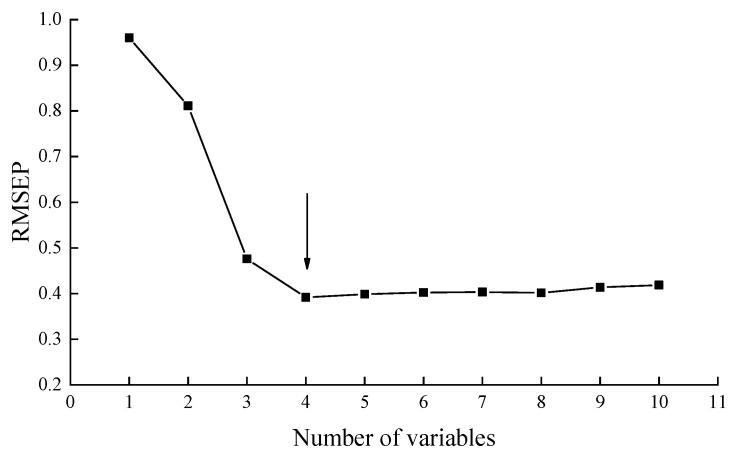
Root mean square error of prediction (RMSEP) of number of variables selected by successive projection algorithm (SPA).

The main purpose of SPA was to select the smallest and most representative variable combination of collinearity. The SPA method was used to solve the collinearity problem existing in the above sensor array and eliminate redundant sensors. Figure 8 shows the root mean square error of the prediction set of variables. When the number of variables was four, the value of RMSEP was relatively the smallest and the model performance was considered to be the best. Consequently, the variable set when the number of variables was four had corresponding sensors W5S, W1W, W2W and W3S. A comprehensive load analysis of the sensors revealed W5S, W1W, W2W and W3S as the final optimized array (featured sensors). This result validated the consistency of the E-nose signal and GC-MS analysis of biofilms.

#### 3.3.4. Quantitative Models

**Figure 9:**
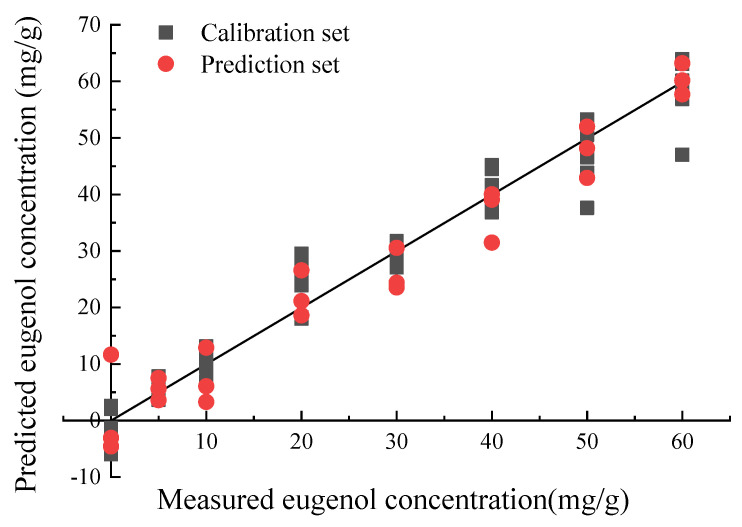
The quantitative prediction models of all sensors based on partial least squares (PLS) algorithms for the determination of eugenol concentration in curdlan (CD) biofilms.

The prediction models of all and featured sensors based on PLS and SVM algorithms for the determination of eugenol concentration in biofilms were established using 80 samples (calibration set: prediction set = 7:3) after pre-processing using the auto scale algorithm. The number of latent variables (LVs) of PLS was 10. The SVM model adopted for the study entailed mainly the use of the radical basis function as the SVM kernel type, which has the loss function epsilon (0.01), the penalty coefficient C (100) and the kernel factor γ (0.0316). The results of the model prediction are shown in Table 3, which indicate that all models are effective for predicting eugenol concentrations (R^2^_p_ > 0.89, RPD > 3). In terms of effectiveness of the modeling methods, the PLS model was superior to SVM in predicting eugenol concentration based on both all sensors and featured sensors. The prediction effect of featured sensors modeling in SVM prediction model was better than that based on all sensors, but the prediction effect in PLS model was contrary. It indicated that the SPA algorithm might eliminate a small amount of effective information while reducing redundant information, thus affecting the accuracy of the model. The number of E-nose sensors is smaller than the number of spectral wavelengths, and this enhances the speed or rapidity of the prediction model based on all sensors. Therefore, the prediction model of eugenol concentration in biofilms based on all sensors is more reliable to retain the original information and improve the prediction accuracy. In summary, the optimal prediction model for eugenol concentration in biofilms was achieved by the PLS prediction model based on all sensors, with R^2^_p_ of 0.952 and RMSEP of 4.612 mg/g (Figure 9). The R^2^_p_ in this experiment was close to or even higher than the values reported in the literature [35,36], which proved that the quantitative prediction model based on E-nose for eugenol in biofilms was effective.

## 4. Conclusions

This paper described a rapid, non-destructive quantitative method for determining eugenol concentration in biofilms based on the E-nose technique combined with GC-MS. The results showed a correlation between E-nose sensor characteristics and the different volatile compounds identified by the GC-MS. However, the eugenol concentration determined by GC-MS analysis in higher concentration films (from E-20) and above tended to be saturated, hence it could not be quantified. Also, a qualitative discriminant model for rapid semi-quantitative evaluation of eugenol concentration in biofilms was developed based on PCA and LDA. Finally, the optimal quantitative prediction model for eugenol concentration based on PLS using 10 sensors was established with an R^2^_p_ of 0.952. The method is applicable for screening a large number of samples and represents a new approach for the detection of volatile additives in bio-based packaging materials.

## Figures and Tables

**Table 1:** Eugenol concentrations in biofilms with different eugenol concentrations in film-forming solution (FFS) (n = 3).

Samples	Eugenol Concentration in FFS (mg/g)	Eugenol Concentration in Biofilm (mg/g)
E-5	5	0.782 ± 0.056 g
E-10	10	1.181 ± 0.245 f
E-20	20	2.053 ± 0.300 e
E-30	30	2.586 ± 0.268 d
E-40	40	4.617 ± 0.150 c
E-50	50	5.381 ± 0.349 b
E-60	60	6.476 ± 0.334 a

Note: The data with different letters (a–g) in the same column are significantly different (*p* < 0.05). Values: mean ± standard deviation.

**Table 2:** Volatile compounds of biofilm samples with different eugenol concentration tested by gas chromatography-mass spectrometry (GC-MS).

Peak No.	Volatile Compounds	Relative Concentration (Mean ± SD) %							
		E-0	E-5	E-10	E-20	E-30	E-40	E-50	E-60
Alkane									
1	Pentadecane, 2,6,10,14-tetramethyl-	0.334 ± 0.029 b	0.466 ± 0.045 a	0.217 ± 0.031 c	0.144 ± 0.007 d	0.241 ± 0.010 c	0.189 ± 0.003 cd	0.231 ± 0.023 c	0.184 ± 0.017 cd
2	Tetradecane	0.543 ± 0.105 a	0.207 ± 0.012 b	0.191 ± 0.057 b	0.107 ± 0.008 b	0.130 ± 0.027 b	0.134 ± 0.013 b	0.095 ± 0.014 b	0.131 ± 0.013 b
3	Hexadecane	0.453 ± 0.189 a	0.485 ± 0.212 a	0.239 ± 0.016 ab	0.279 ± 0.132 ab	0.267 ± 0.045 ab	0.187 ± 0.033 ab	0.227 ± 0.006 ab	0.150 ± 0.015 b
4	Dodecane	0.494 ± 0.060 a	0.165 ± 0.106 b	0.119 ± 0.010bc	0.031 ± 0.004 c	0.045 ± 0.038 c	0.027 ± 0.006 c	0.018 ± 0.002 c	0.025 ± 0.005 c
5	Eicosane	0.476 ± 0.375	0.100 ± 0.034	0.139 ± 0.013	N.D.	0.040 ± 0.14	0.015 ± 0.004	N.D.	0.047 ± 0.025
6	Heptadecane	0.106 ± 0.006 b	0.264 ± 0.067 a	0.111 ± 0.018 b	0.119 ± 0.025 b	0.160 ± 0.001 b	0.094 ± 0.002 b	0.142 ± 0.044 b	0.137 ± 0.014 b
7	Tridecane	0.819 ± 0.004 a	0.245 ± 0.048 b	0.185 ± 0.023 c	0.065 ± 0.007 d	0.061 ± 0.032 d	0.048 ± 0.001 d	0.034 ± 0.007 d	0.031 ± 0.002 d
8	Octadecane	0.132 ± 0.010 a	0.085 ± 0.031 b	0.054 ± 0.011 bc	0.042 ± 0.004 c	0.058 ± 0.005 bc	0.048 ± 0.017 c	0.037 ± 0.012 c	0.044 ± 0.005 c
9	Cyclopentadecane	0.336 ± 0.014	0.181 ± 0.032	N.D.	0.046 ± 0.034	N.D.	0.018 ± 0.001	0.037 ± 0.003	0.026 ± 0.008
	∑	2.796 ± 0.762 a	2.198 ± 0.254 a	1.185 ± 0.119 b	0.834 ± 0.191 b	0.982 ± 0.010 b	0.744 ± 0.053 b	0.802 ± 0.104 b	0.762 ± 0.076 b
Olefin									
10	1-Octadecene	0.318 ± 0.024 a	0.215 ± 0.123 ab	0.067 ± 0.005 b	0.179 ± 0.025 ab	0.263 ± 0.118 a	0.072 ± 0.031 b	0.070 ± 0.054 b	0.083 ± 0.006 b
11	Naphthalene, 1,2,3,4-tetrahydro-1,6-dimethyl-4-(1-methylethyl)-, (1S-cis)-	0.173 ± 0.068 ab	0.195 ± 0.003 a	0.129 ± 0.026 abc	0.088 ± 0.026 bc	0.123 ± 0.014 abc	0.135 ± 0.048 abc	0.071 ± 0.003 c	0.051 ± 0.004 c
12	D-Limonene	0.412 ± 0.072	0.186 ± 0.011	0.067 ± 0.005	N.D.	N.D.	N.D.	N.D.	N.D.
13	Caryophyllene	N.D.	0.050 ± 0.036	0.085 ± 0.020	0.022 ± 0.004	0.079 ± 0.026	0.063 ± 0.020	0.028 ± 0.002	0.134 ± 0.023
14	2-Tetradecene, (E)-	N.D.	0.081 ± 0.019	0.059 ± 0.003	N.D.	N.D.	N.D.	N.D.	N.D.
15	1-Hexadecene	N.D.	0.081 ± 0.005	0.130 ± 0.078	0.020 ± 0.003	N.D.	N.D.	N.D.	N.D.
16	alpha-caryophyllene	N.D.	0.205 ± 0.019	0.203 ± 0.008	N.D.	N.D.	0.224 ± 0.021	N.D.	N.D.
17	Caryophyllene oxide	N.D.	N.D.	N.D.	0.065 ± 0.012	0.149 ± 0.077	0.065 ± 0.016	N.D.	0.091 ± 0.002
	∑	0.903 ± 0.060 a	0.817 ± 0.083 ab	0.742 ± 0.073 ab	0.364 ± 0.053 de	0.613 ± 0.155 bc	0.445 ± 0.179 cd	0.154 ± 0.038 e	0.359 ± 0.036 de
Aldehyde									
18	Heptanal	1.192 ± 0.418	0.141 ± 0.176	0.064 ± 0.064	N.D.	N.D.	N.D.	N.D.	N.D.
19	2-Octenal, (E)-	1.976 ± 0.521 a	0.448 ± 0.210 b	0.214 ± 0.028 b	0.066 ± 0.014 b	0.046 ± 0.012b	0.048 ± 0.003b	0.054 ± 0.013b	0.022 ± 0.008b
20	Decanal	0.488 ± 0.246	0.223 ± 0.065	0.160 ± 0.020	0.062 ± 0.007	0.035 ± 0.006	N.D.	N.D.	0.017 ± 0.002
21	Nonanal	1.379 ± 0.456 a	0.566 ± 0.070 b	0.470 ± 0.012 bc	0.097 ± 0.024 bc	0.052 ± 0.004 bc	0.101 ± 0.015 bc	0.086 ± 0.033 bc	0.024 ± 0.003 c
22	2-Nonenal, (E)-	2.462 ± 0.657 a	0.614 ± 0.212 b	0.385 ± 0.078 b	0.160 ± 0.039 b	0.126 ± 0.055 b	0.089 ± 0.014 b	0.092 ± 0.023 b	0.050 ± 0.002 b
	∑	7.497 ± 2.109 a	1.994 ± 0.733 b	1.293 ± 0.179 b	0.385 ± 0.006 b	0.259 ± 0.045 b	0.237 ± 0.032 b	0.233 ± 0.068 b	0.095 ± 0.013 b
Aromatic compound									
23	Eugenol	N.D.	51.770 ± 9.310	77.086 ± 10.167	89.330 ± 1.268	86.498 ± 3.363	81.465 ± 6.145	88.700 ± 1.010	86.952 ± 0.909
24	Benzene, 1,2-dimethoxy-4-(2-propenyl)-	0.192 ± 0.056 b	0.136 ± 0.014 b	0.209 ± 0.001 b	0.242 ± 0.037 b	0.419 ± 0.087 a	0.423 ± 0.025 a	0.164 ± 0.026 b	0.221 ± 0.063 b
25	Phenol, 2-methoxy-4-(1-propenyl)-, (E)-	2.586 ± 1.683 a	2.966 ± 0.140 a	3.461 ± 0.216 a	2.873 ± 0.230 a	2.456 ± 0.015 a	2.469 ± 0.409 a	2.076 ± 0.212 a	1.876 ± 0.007 a
26	Naphthalene	0.137 ± 0.004 a	0.070 ± 0.024 b	0.035 ± 0.010 c	0.032 ± 0.003 c	0.039 ± 0.023 c	0.021 ± 0.003 c	0.019 ± 0.003 c	0.027 ± 0.013 c
27	1,1’-Biphenyl, 2,2’,5,5’-tetramethyl-	N.D.	N.D.	N.D.	N.D.	N.D.	0.048 ± 0.002	0.088 ± 0.004	N.D.
	∑	2.824 ± 1.568 c	53.459 ± 11.417 b	80.773 ± 9.974 a	92.477 ± 1.463 a	88.184 ± 8.461 a	84.425 ± 6.584 a	91.005 ± 1.132 a	89.076 ± 0.841 a

Note: The data without same letters (a–c) in the same row are significantly different (*p* < 0.05). Values: mean ± standard deviation. N.D., not detected.

**Table 3:** Prediction models of all sensors and characteristic sensors of eugenol concentration based on partial least squares (PLS) and support vector machines (SVM) algorithm.

Modeling Method	Number of Variables	R^2^_c_	R^2^_p_	Root Mean Square Error of Prediction (RMSEP)/(mg/g)	Residual Predictive Deviation (RPD)
PLS	10	0.962	0.952	4.612	4.530
	4	0.955	0.948	4.706	4.440
SVM	10	0.998	0.897	6.613	3.159
	4	0.990	0.905	6.327	3.302
